# Development of the shear displacement of sandy soil due to absorption under constant shear stress for creep failure

**DOI:** 10.1038/s41598-022-19287-1

**Published:** 2022-09-05

**Authors:** Katsuo Sasahara

**Affiliations:** Kochi University, 2-5-1, Akebonocho, Kochi, 780-8520 Japan

**Keywords:** Geophysics, Civil engineering, Natural hazards

## Abstract

Measurement of the displacement and pore pressure in physical model slopes and natural slopes revealed that not only an accelerative increase up to failure but also a decelerative increase occurred in surface displacement under a constant groundwater level, which could be recognized as creep deformation under constant stress. The displacement increased significantly at first in both types, which made it difficult to evaluate whether the displacement developed to the point of failure at the start of the increase. It was necessary to find an indicator for evaluation as the first step of the prediction of an onset of slope failure. Measurement of shear and normal displacements of the sandy specimen in an inclined direct shear box with increasing water content was conducted to examine the indicator. The increase in the shear displacement was categorized into three types: the accelerative increase up to failure with increasing volumetric water content (VWC), the accelerative increase up to failure under constant VWC just prior to failure, and the decelerative increase under constant VWC. It was recognized that a constant VWC corresponded to constant suction from the experimental data. Shear displacement increased up to failure with the monotonic decrease in void ratio just prior to failure in the case of increasing VWC. The void ratio monotonically decreased under constant VWC in the case of shear displacement termination under constant VWC, while it significantly varied at first and then converged to a constant value just prior to failure in the case of shear displacement increase with constant VWC. The ultimate void ratio under the same stress conditions might have been unique. These facts revealed that the void ratio can be recognized as an indicator of the failure of specimens under creep deformation according to absorption. When the void ratio converges to a constant value under constant VWC, the shear displacement might increase up to failure, while the void ratio varying under constant VWC indicates that the shear displacement terminates before failure.

## Introduction

Many cases of the measurement of displacement and pore pressure in natural slopes or physical model slopes have been conducted to reveal the mechanism of an onset of a rainfall-induced landslide^1–8^. The displacement increased acceleratively with the increase in the groundwater level just prior to failure in some cases^1,2,4,7^, while it increased under a constant groundwater level in other cases^6,8^. The former cases are recognized as the shear displacement increase due to the increase in stress ratio according to the decrease in effective normal stress. The shear displacement development with increasing pore pressure in a slope was modelled by a hyperbolic relationship between the pore pressure and the shear displacement based on the measured data on a sandy model slope under constant rainfall intensity^4,5^. The latter cases are recognized as creep displacement under constant stress. In particular, an accelerative increase in the displacement under a constant groundwater level could be recognized just prior to failure of a slope in some cases^8^. However, not only the accelerative increase but also the decelerative increase in the displacement were also observed under a constant GWL^6^. The increase in the displacement was significant at first, and then the ratio of the increase in the displacement decreased until the termination of the displacement in this case. A decelerative increase and accelerative increase in the displacement under constant stress were also observed in the model slope under multistep excavation^9^. Both increases developed after the excavation in this experiment, which indicates that these were creep displacements under constant stress conditions. Accelerative increase developed up to failure, while decelerative increase terminated at some time and did not reach failure. The displacement increases significantly at the start of the decelerative increase, so it is difficult to evaluate whether the displacement develops up to failure or not at the start of both increases.

The displacement under constant stress conditions is recognized as creep deformation and modelled as time-dependent deformation. Christensen and Wu^10^, Murayama and Shibata^11^, Komamura and Huang^12^ and Ter-Stepanian^13^ developed a rheological model to explain the creeping shear behaviour of clayey soil. Sekiguchi and Ohta^14^, Adachi and Oka^15^, Desai et al.^16^, Wheeler et al.^17^, Dafalias et al.^18^, and Leoni et al.^19^ proposed elasto-viscoplastic models to simulate time-dependent shear deformation under anisotropic conditions. These models could explain the time variation in the displacement under constant shear stress, i.e., accelerative and decelerative increase with time, but could not evaluate whether the displacement increased to the point of failure at the start of the displacement.

Murayama and Shibata^11^ and Bhat^20,21^ examined the condition for the start of the increase in the displacement up to failure for creep deformation in triaxial compression tests or ring shear tests under constant shear stress. They reported that accelerative displacement occurred under constant shear stress above the residual shear strength in the case of clayey soil. However, the condition for decelerative displacement has not yet been examined. Moreover, conditions for the accelerative and decelerative increase in the displacement under constant stress for sandy soil, which is the main material of natural slopes in Japan^22^, have not been examined.

When the measurement at an actual natural slope is conducted for early warning against slope failure, the shear strength should be measured by indoor shear tests before the measurement to adopt their theories. It is rather difficult to know the shear strength before the measurement because they need much labour and time. Instead of the shear strength, Sasahara et al.^23^ adopted the normal displacement versus shear displacement as the indicator to know the instability of the model slope. The normal displacement approached a constant value with the increase in the shear displacement on the surface of the model slope. The idea comes from the principle of dilatancy of sand in direct shear conditions, in which the ratio of normal strain to shear strain approaches zero at steady state just prior to failure of the soil. The above idea requires only multi-dimensional measurement of displacement on the slope in real time without the shear strength of a soil. It is very convenient for the measurement in actual slopes for early warning against landslides.

In this paper, the shear and normal displacements of the specimen in an inclined direct shear box with increasing water content under constant anisotropic stress were measured to examine the possibility of evaluating the condition of the development in the shear displacement up to failure in the direct shear condition based on the idea of dilatancy.

## Methodology

### Test apparatus and material

Shear deformation of the soil layer in an infinite slope according to rainfall infiltration can be recognized as simple shear under almost constant shear stress with the increase in the volumetric water content. In particular, the deformation of a soil mass along the slip surface can be modelled as direct shear. The weight of the soil mass increases slightly as the volumetric water content increases, and it causes an increase in not only shear stress but also normal stress on the soil mass near the slip surface under unsaturated conditions, which results in an almost constant stress ratio on the slip surface.

**Figure 1:**
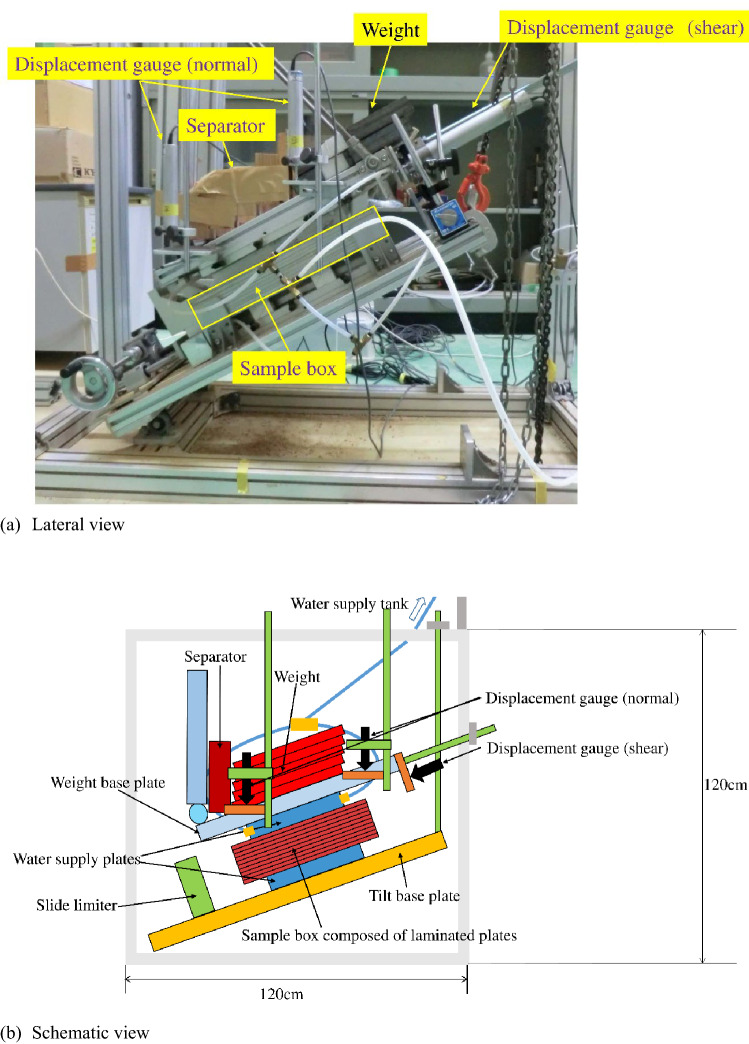
Inclined simple shear box.

**Figure 2:**
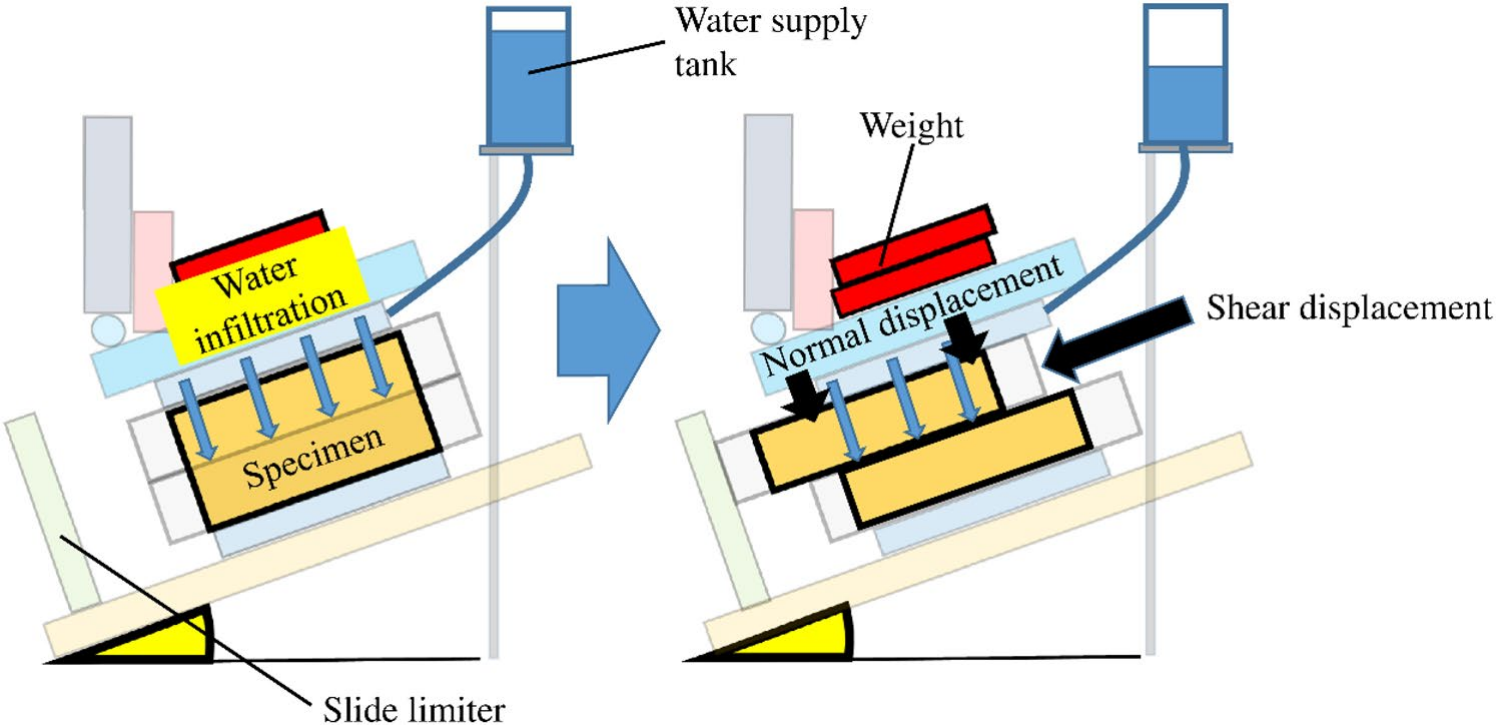
Schematic diagram of the shear deformation of the specimen according to the water supply.

**Figure 3:**
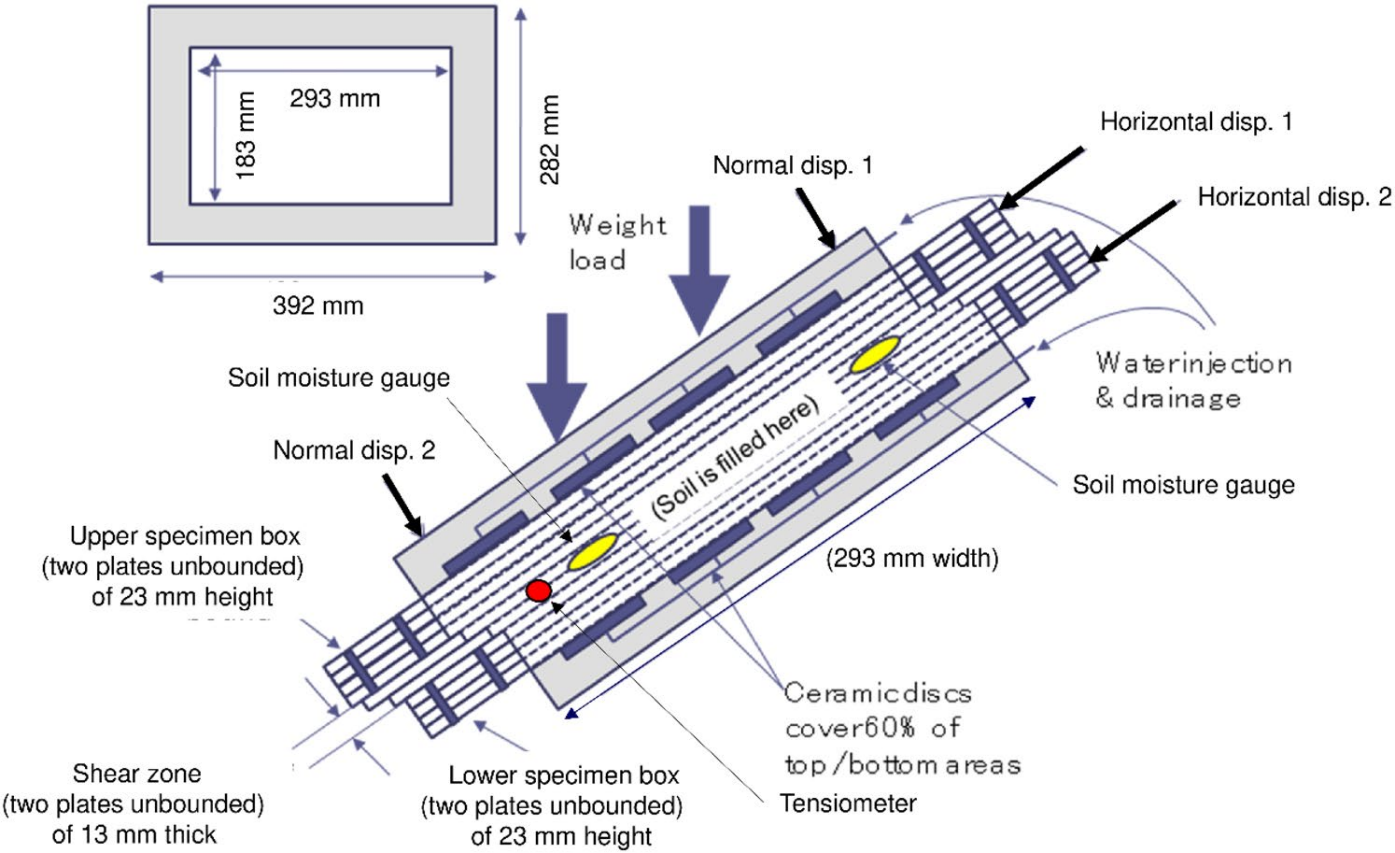
Upper and lower specimen boxes.

An inclined simple shear box (Fig. 1) was adopted to simulate the shear deformation in the slope according to rainfall infiltration. Figure 2 is the diagram of the shear deformation of the specimen according to the water supply. Water is supplied from the water supply tank above the apparatus, and it infiltrates into the specimen, which results in an increase in the volumetric water content (hereafter VWC) in the specimen. The specimen is inclined on the tilt base plate to apply shear stress, and normal stress is loaded by the weights. The specimen is divided into upper and lower parts and has a thin shear zone at the middle of the direct shear box. The upper specimen displaces downwards along the inclined shear zone according to the increase in the VWC until the slide limiter. Shear displacement is measured by the displacement gauge at the front and back sides at the upper boundary of the upper specimen box, while normal displacement is measured by upper and lower displacement gauges at the surface of the upper specimen box, as shown in Fig. 1. The displacement gauges for shear displacement and for normal displacement are fixed at the flame of the apparatus and supporting rods from the flume, respectively. The resolutions of the displacement gauges for shear and normal displacement were 0.1 mm and 0.05 mm, respectively. The specimen box is composed of laminated plates (Fig. 3). Aluminium plates that are 5 mm thick and Teflon plates that are 1 mm thick are stacked alternately to make the specimen box. Four aluminium and three Teflon plates are bounded to make the upper and lower specimen boxes solid. Two aluminium and three Teflon plates between the specimen boxes are free to make a shear zone. The soil specimens in the boxes composed of laminated plates are 293 mm wide, 183 mm long and 59 mm high. The height of the shear zone made of free plates is 13 mm. Two soil moisture sensors with a resolution of 3% of the VWC are inserted at the shear zone, as shown in Fig. 3. A tensiometer with a solution of 0.5 kPa is also inserted at the shear zone to measure the suction in some cases. Water is supplied to the specimen through the ceramic disc with an air entry value of 100 kPa, as shown in Fig. 3, because the soil specimen is unsaturated. The total area of the ceramic disc is 60% of the area of the upper and lower water supply plates. The area of the water supply plates is slightly smaller than that of the specimen, which makes it possible to let the upper water supply plate move vertically to the specimen in accordance with the compression and dilation of the specimen. The movement is measured by displacement gauges as the normal displacement of the specimen.

The procedure of the preparation and implementation of the experiment is as follows.

1. Laminated plates are piled up and bounded to make upper and lower specimen boxes on the lower water supply plate fixed on the horizontal tilt base plate.
2. The soil is poured and compacted to make a specimen with a targeted void ratio in the specimen boxes.
3. The upper water supply plate is placed beneath the weight base plate on the specimen.
4. We place weights on the weight base plate and install shear and normal displacement gauges to contact targets fixed to the weight base plate. The targets can move following the displacement of a part of the specimen in the upper specimen box.
5. The tilt base plate is inclined to the targeted inclination of the slope.
6. The specimen is left for self-weight consolidation for approximately 12 h.
7. Water supply is started (start of experiment).

**Figure 4:**
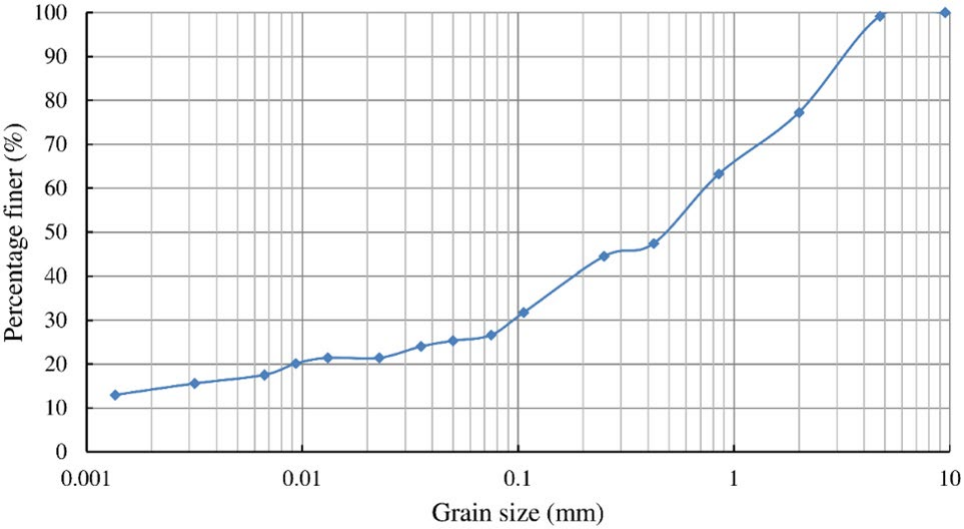
Grain size distribution of the soil for the specimen.

The grain size distribution and physical properties of the soil used for the specimens are shown in Fig. 4 and Table 1. The grain size distribution, specific gravity of the soil minerals, and maximum and minimum void ratios were analysed based on JIS A 1204, JIS A 1202 and JIS A 1224, respectively^24^. Note that the method for the analysis of maximum and minimum void ratio, JIS A 1244, should be adopted only for the soil with less than 5% of fine material (grain size was less than 0.075 mm), while the soil used for this study violated this condition and had more fine material. In particular, the minimum void ratio of the soil with much fine material measured in this method is often analysed to be larger than the actual value under confining pressure. While the relative density can be derived from the maximum and minimum void ratios to evaluate the degree of looseness of the soil. No other method could be found to evaluate the degree of looseness of the soil, thus the standard method JIS A 1244 was adopted in this study.

**Table 1:** Physical soil properties of the soil for the specimen.

Specific gravity of soil mineral *G*_S_	2.481
Maximum void ratio of the soil *e*_max_	1.31
Minimum void ratio of the soil *e*_min_	0.9
60% particle size *d*_60_	0.85
30% particle size *d*_30_	0.093
10% particle size *d*_10_	0.037
Coefficient of uniformity *U*_C_	23

### Experimental conditions

Five series of experiments, which were the series for the examination of repeatability, effect of rainfall intensity, effect of normal stress, and effect of inclination of the slope, were implemented, as shown in Table 2. The series for the repeatability was implemented for four cases with the same condition, which means the same initial condition of the specimen, the same water head (1500 mm high from the bottom of the water supply tank to the centre of the specimen), the same load of weights, and the same inclination of the slope. The series for the examination of the effect of rainfall intensity included two series, namely, “Rainfall intensity 1” and “Rainfall intensity 2”. They were implemented for four cases with different water heads (height from the bottom of the water supply tank to the centre of the specimen). The initial condition of the specimen, the inclination of the slope and the loaded weights were basically the same in these series. The initial VWC at “Rainfall intensity 1” was lower, and the initial void ratio could not be the same. The series of different normal stresses “*σ*_N_” and different inclinations of slope “slope” were implemented for the specimen with the same initial condition under different numbers of weights and different inclinations of the slope, respectively. Each series had two cases with different loading conditions. Oven-dried soil was used to construct specimens in the specimen box, while the VWC increased slightly due to the absorption of water from the ceramic discs at the water supply plates during 12 h of the self-weight compression stage. The specimen was constructed by manual stamping with a targeted void ratio of 0.88, which was the average void ratio of the middle-scale slope model made of the same soil^25^. It was determined that failure of the specimen occurred when the specimen was displaced to the point of contacting the slide limiter.

**Table 2:** Experimental cases.

Series	No	VWC_ini._ (%)	*e*_ini_	*θ* (°)	RI (mm/h)	*σ*_N_ (kN/m^2^)	Failure	Time of Failure (s)	VWC_fin._ (%)	*e*_fin_	Creep	Suction measurement
Reproducibility	No. R1	11.5	0.914	30	3.11	8.61	Yes	17,510	36.3	0.845	Yes	No
	No. R2	10.8	0.929	30	7.09	8.61	Yes	14,160	32.1	0.763	No	No
	No. R3	9.6	0.94	30	3.09	8.61	Yes	18,160	36.3	0.93	Yes	No
	No. R4	10.3	0.878	30	3.29	8.61	Yes	18,170	37.5	0.774	Yes	No
Rainfall intensity 1	No. 1	4.6	0.93	30	3.94	8.61	Yes	14,620	34.3	0.884	Yes	Yes
	No. 2	4	0.946	30	2.79	8.61	Yes	20,700	35.9	0.941	No	Yes
	No. 3	5.1	0.923	30	2.3	8.61	Yes	26,280	35.7	0.872	Yes	Yes
	No. 4	3.2	0.922	30	1.62	8.61	Yes	36,450	34.2	0.823	No	Yes
*σ*_N_	No. 5	2.8	0.913	30	4.14	3.21	Yes	16,650	35.1	0.86	No	Yes
	No. 7	0.7	0.932	30	3.95	14.96	Yes	16,270	35.9	0.935	Yes	Yes
Slope	No. 8	1.8	0.899	25	3.81	9.01	No	-	35.4	0.799	Yes	Yes
	No. 10	3.2	0.915	35	4.05	8.14	Yes	16,150	35.2	0.952	No	Yes
Rainfall intensity 2	No. 11	8.7	0.89	30	11.7	8.61	Yes	10,650	35.3	0.859	Yes	No
	No. 12	7.7	0.905	30	12.6	8.61	Yes	9,530	34	0.756	No	No
	No. 13	8.83	0.928	30	6.15	8.61	Yes	34,690	36.7	0.861	Yes	No
	No. 14	8.39	0.88	30	3.17	8.61	No	-	35	0.852	Yes	No

*VWC*_*ini.*_* (%) *initial volumetric water content, *e*_*ini*._ initial void ratio, *θ (°)* inclination of model slope, *RI * rainfall intensity, *σ*_*N*_^*2*^ normal stress, *VWC*_*fi.*_* (%) *final volumetric water content, *e*_*fin*._ final void ratio.

## Results

### Reproducibility of the test

**Figure 5:**
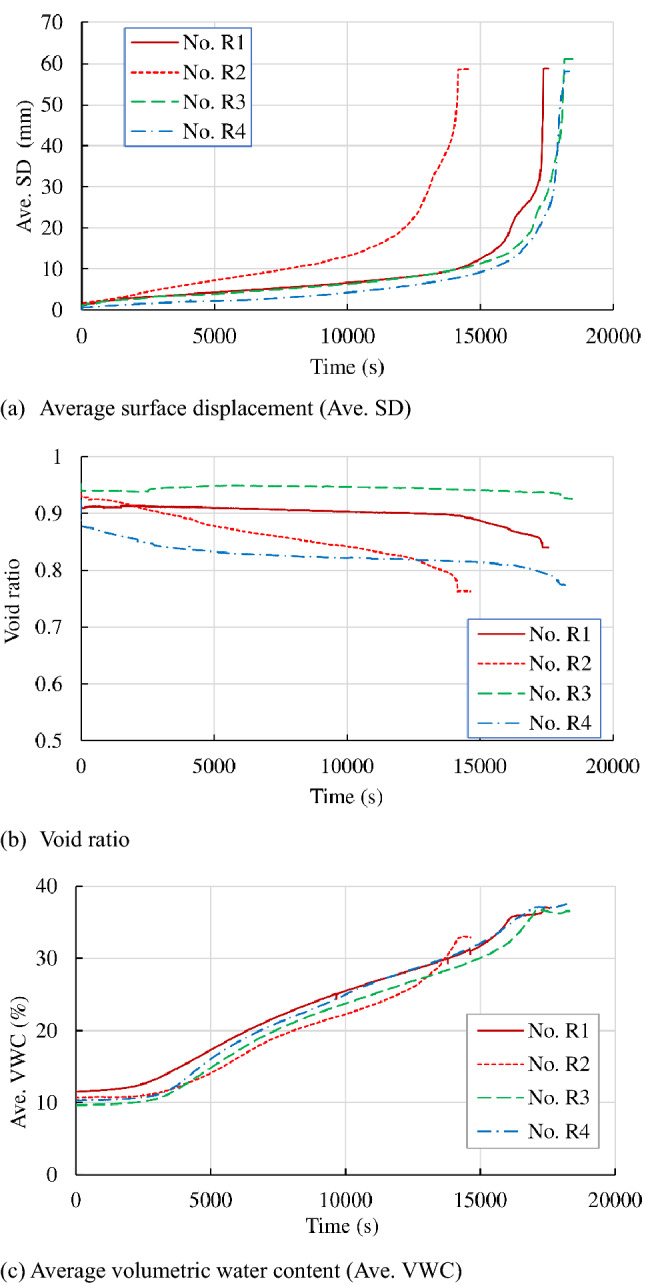
Temporal variation in the average surface displacement, void ratio and average volumetric water content in the “reproductivity” series.

Figure 5 shows the results of the tests in the series of “Reproducibility” to examine the reproducibility of the test. Figure 5a–c indicate temporal variations in the average shear displacement (Ave. SD), void ratio and average VWC (Ave. WC), respectively. The average shear displacement and average VWC are the averages of the measured displacements and VWCs by two displacement gauges and two soil moisture gauges, respectively. The void ratio is derived from the initial height of the specimen and the average normal displacement (Ave. ND), which is the average of the measured normal displacement by two displacement gauges. Figure 5a indicates that the temporal variation in average shear displacement was almost identical in the cases under the same rainfall intensity (Nos. R1, R3, and R4), while the shear displacement proceeded faster in the case (No. R2) under a larger rainfall intensity. Nos. R3 and R1 with 0.94 and 0.914 void ratios, respectively, showed almost constant void ratios at first and then decreased prior to failure, while the void ratio monotonically decreased in No. R2, which had almost the same initial void ratio but higher rainfall intensity, as shown in Fig. 5b. No. R4 with a lower initial void ratio showed a monotonic decrease in the void ratio. The slope of the temporal variation in the void ratio basically depends on the initial void ratio but also on the rainfall intensity. Figure 5c indicates that the temporal variation in the average VWC was almost identical, with some scatter in Nos. R1, R3 and R4 under almost the same rainfall intensity, while the increase in the VWC was faster prior to failure in No. R2 with higher rainfall intensity.

The shear displacement and VWC indicated almost identical temporal variations under the same initial conditions and same rainfall intensity, while the temporal variation in the void ratio was much influenced by the initial void ratio.

### VWC and suction

**Figure 6:**
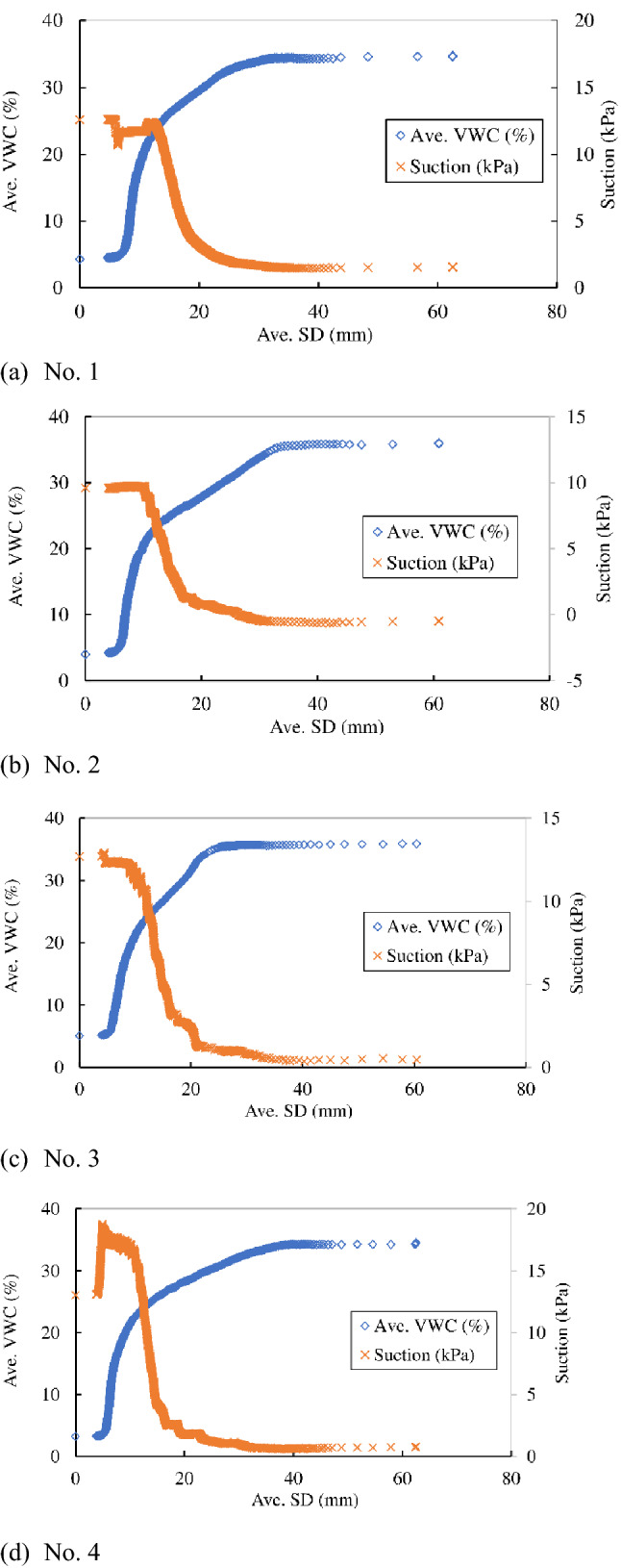
Variation in average volumetric water content (Ave. VWC) and suction along the average surface displacement (Ave. SD) in Nos. 1, 2, 3 and 4.

Figure 6a–d indicate the variation in average VWC and suction along average shear displacement to compare the variation of VWC to suction in Nos. 1, 2, 3 and 4 in the series of “Rainfall intensity 1”, respectively. Average shear displacement was adopted instead of time to focus on the behaviour over a large displacement range prior to failure. The results indicate that the average VWC increased at first and then stayed constant just prior to failure. The suction decreased significantly at first and then remained constant. The shear displacement at the start of constant suction was slightly smaller than that at the start of constant VWC, which suggests that the suction reached a constant value slightly faster than the VWC. Thus, displacement under constant VWC corresponds to creep displacement under constant stress conditions in this test.

**Figure 7:**
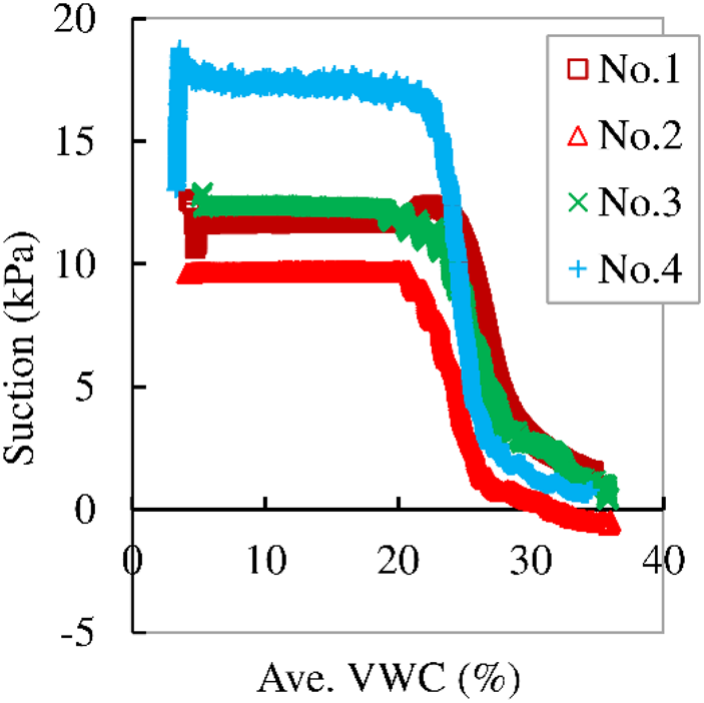
Relationships between the average volumetric water content (Ave. VWC) and suction in Nos. 1, 2, 3 and 4.

Figure 7 indicates the relationships between the average VWC and suction in Nos. 1, 2, 3and 4 in the series of “Rainfall intensity 1”. The suction remained almost constant from the start of the experiment to around 20% of average VWC in each case. It might have been due to the insufficient duration for the equalization of the suction between the porous cup of the tensiometer and the soil. The suction in the soil might have been higher than the measured value by the tensiometer in this region of average VWC. The suction decreased with the increase in average VWC beyond around 20% of average VWC. The trend of the decrease in the suction with average VWC was almost identical with around 5 kPa of the fluctuation of the suction in each case. Maximum of average VWC was around 35% with the final suction between − 0.5 to 1.5 kPa. Considering the resolution of tensiometer (0.5 kPa), the soil was quasi-saturated at maximum average VWC event though no back pressure was loaded to the specimen.

### Shear and normal displacement, VWC in the tests

**Figure 8:**
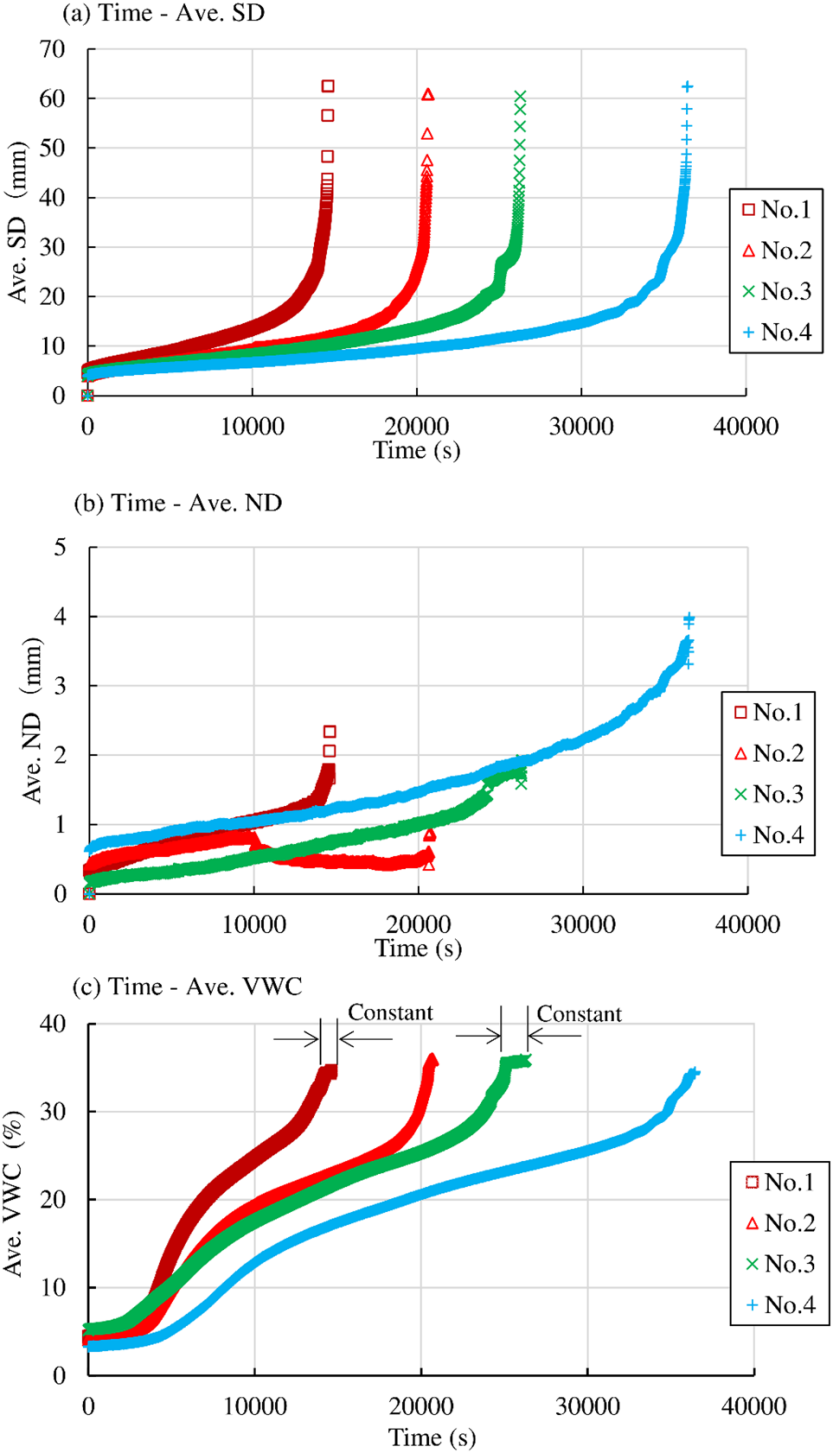
Temporal variation in the average surface displacement (Ave. SD), average normal displacement (Ave. ND) and average volumetric water content (Ave. VWC) in Nos. 1, 2, 3 and 4 in the series of “Rainfall intensity 1”.

Temporal variation in Average surface displacement (Ave. SD), average normal displacement (Ave. ND), and average volumetric water content (Ave. VWC) in Nos. 1, 2, 3 and 4 in the series of “Rainfall intensity 1” are shown in Fig. 8a–c, respectively. Rainfall intensity in No.1 was highest and it decreased with the number of the case according to Table 2, while the initial VWC and void ratio were 3–5% and 0.92–0.05, respectively. The initial VWC and void ratio could have been recognized as almost same in this series. Ave. VWC showed small increase until 3,000–4,000 s at first and then increased significantly until failure. Ave. VWC monotonically increased up to failure in Nos. 2 and 4, while it increased and then stayed constant just prior to failure in Nos. 1 and 3. Figure 6 indicated that the constant VWC suggested constant suction. Ave. SD indicated accelerative increase with time up to failure in each case. The case with smaller rainfall intensity needed more time up to failure in this series. It suggested that rainfall intensity influenced much to failure of the slope. Ave. SD increased with increasing VWC up to failure in Nos. 2 and 4, while it increased with increasing VWC at first and then increased under constant VWC just prior to failure in Nos. 1 and 3. Ave. ND indicated gradual increase (compression) with the increase in Ave. SD and then acceleratively increased just prior to failure in Nos. 1, 3 and 4. It gradually increased at first and then dropped down at around 10,000 s in No. 2. It stayed almost constant until 20,000 s and jumped up to failure. The drop at around 10,000 s was error due to inadequate movement of the needle of the displacement gauge while the normal displacement was recognized visually to be measured correctly after that. Thus, specimen indicated compression up to failure also in No.2.

**Figure 9:**
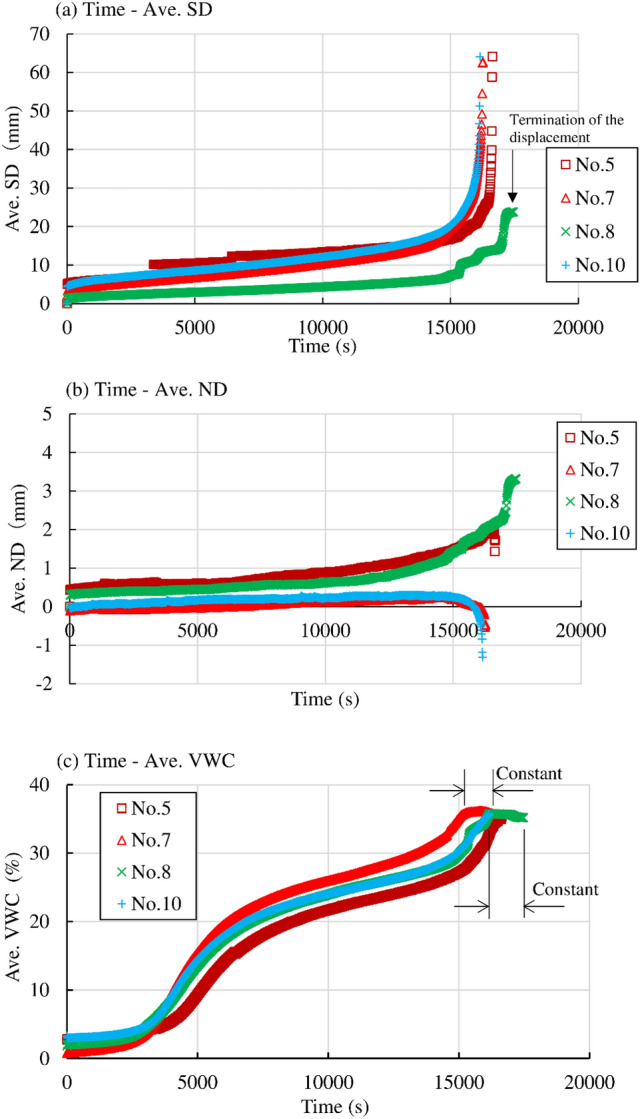
Temporal variation in the average surface displacement (Ave. SD), average normal displacement (Ave. ND) and average volumetric water content (Ave. VWC) in Nos. 11, 12, 13 and 14 in the series of “Rainfall intensity 2”.

Temporal variation in Ave. SD, Ave. ND, and Ave. VWC in Nos. 11, 12, 13 and No. 14 in the series of “Rainfall intensity 2” are shown in Fig. 9a–c, respectively. Rainfall intensity was higher (11.7 and 12.6 mm/h) in Nos. 11 and 12 while it was lower (6.15 and 3.17 mm/h) in Nos. 13 and 14. Initial VWC and initial void ratio were around 8–9% and around 0.88–0.93, which could be recognized as almost same. Ave. VWC showed small increase at first and then increased significantly in each case. It stayed almost constant just prior to failure or termination of the increase in Ave. SD in Nos. 11, 13 and 14 while it monotonically increased up to failure in No. 12. Ave. SD showed accelerative increase in No. 12 while it showed accelerative increase at first and then it showed decelerative increase and finally accelerative increase up to failure under constant average VWC in Nos. 11 and 13. Combination of decelerative and accelerative increase in Nos. 11 and 13 exhibited a progression of primary, secondary and tertiary creep (Saito 1965). Ave. SD gradually increased at first and then terminated in non-failure under constant VWC in No.14. Ave. SD significantly increased up to failure at around 10,000 s in Nos. 11 and 12 with higher rainfall intensity (around 11–13 mm/h) while it increased at failure at around 35,000 s in No. 13 with smaller rainfall intensity (6.15 mm/h). Specimen could not be failed in No. 14 with smallest rainfall intensity. These facts also suggested large influence of rainfall intensity to failure of the slope such as in the series of “Rainfall intensity1”. Ave ND showed gradual increase with the increase in Ave. SD until failure or termination of the displacement in each case. The fact indicated the specimens were compressed with the shear displacement.

**Figure 10:**
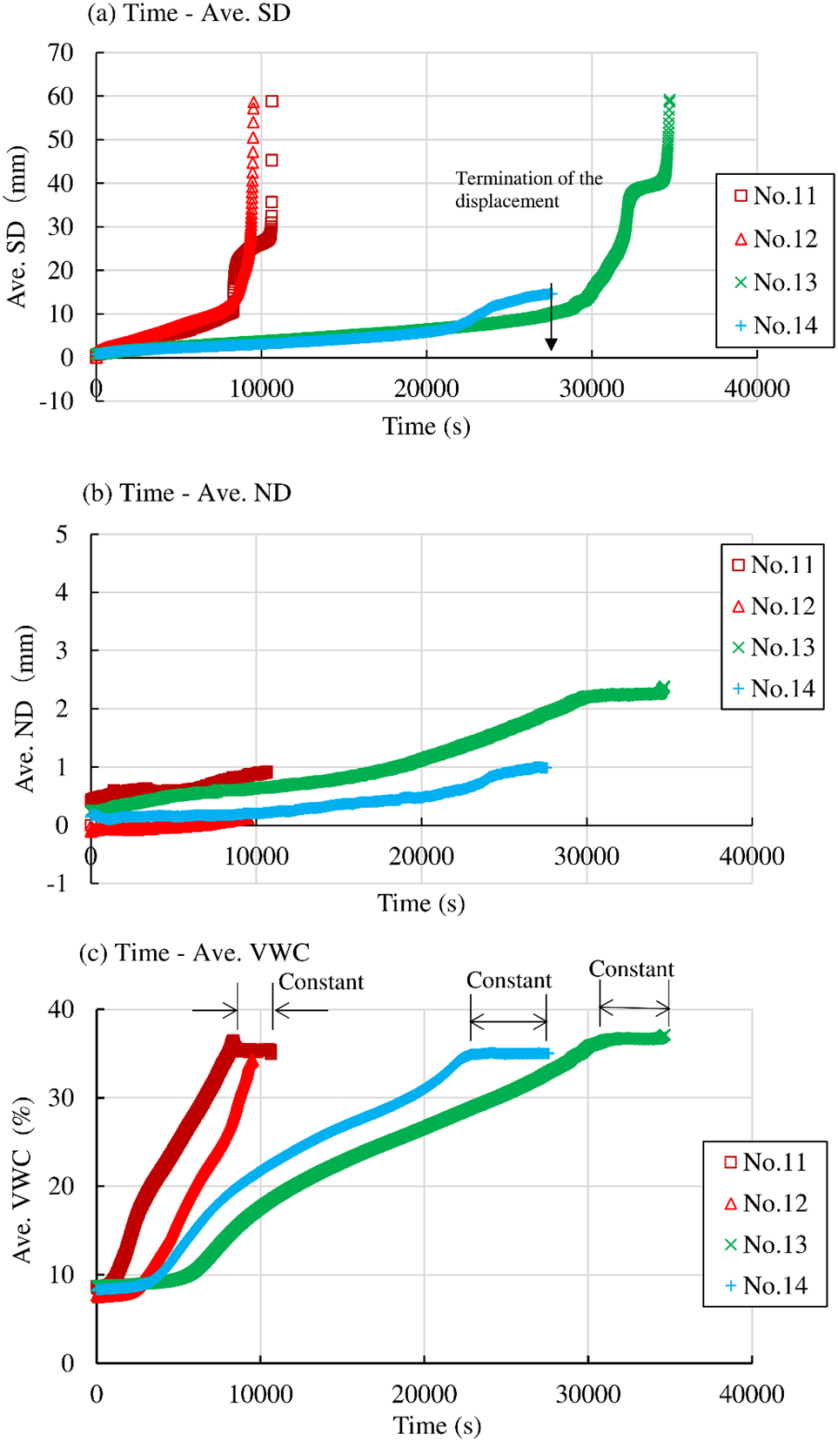
Temporal variation in the average surface displacement (Ave. SD), average normal displacement (Ave. ND) and average volumetric water content (Ave. VWC) in Nos. 5, 7, 8 and 10 in the series of “*σ*_N_” and “slope”.

Temporal variation in Ave. SD, Ave. ND, and Ave. VWC in Nos. 5 and 7 in the series of “*σ*_N_ “, Nos. 8 and 10 in the series of “slope” are shown in Fig. 10a–c, respectively. The normal stress *σ*_N_ at No. 5 are smaller than No. 7 under almost same initial void ratio (0.91–0.93) and same rainfall intensity (around 4 mm/h). Initial VWC in No. 5 was a little bit larger than that in No.7. Ave. VWC increased faster in No.7 than No. 5. It stayed almost constant just prior to failure in No.7 while it monotonically increased up to failure in No.5. Ave. SD identically increased up to failure at 16,150–16,600 s both in Nos. 5 and 7. It developed up to failure under constant VWC just prior to failure in No. 7. These facts suggested that small influence of normal stress could be recognized on shear displacement. Ave. ND monotonically increased just prior to failure and decrease during failure in No. 5 while it stayed almost constant until 15,000 s and decreased to failure in No. 7. Specimen demonstrated compression just prior to failure in No. 5 while it demonstrated small dilation only just prior to failure in No. 7. The fact suggested that specimen tends to dilate with the increase in shear displacement under larger normal stress. Inclination of model slope at No. 10 was higher (35°) than other cases while the inclination in No. 8 was lower (25°) than other case. Initial VWC at No. 10 was a little bit larger than No. 8 while initial void ratio and rainfall intensity was almost identical in both cases. The increase in Ave. VWC with time indicated almost identical until maximum VWC in both cases. The specimen failed at maximum VWC in No. 10 while the displacements developed after the maximum VWC under almost constant VWC until the termination of the displacements in No. 8. Ave. SD showed accelerative increase up to final in both cases. Ave. SD developed faster in No. 10 while it terminated without failure in No. 8. Ave. ND stayed almost constant until 15,000 s and then decreased (dilation) up to failure in No. 10 while it monotonically increased (compression) until the termination of the displacements in No. 8. These facts suggested higher stress ratio with higher inclination of the slope give large influence to shear deformation of the slope. The shear displacement increased faster, and the specimen indicated dilative trend in the case of larger inclination of the slope.

It was found that some of the specimens were deformed under almost constant VWC (suction) just prior to failure. The deformation could be recognized as creep deformation because constant VWC meant constant suction. The specimens in Nos. 1, 3, 7, 8, 11 and 13 demonstrated creep deformation just prior to failure. While the shear displacement increased only with a monotonic increase in VWC until failure in Nos. 2, 4, 5, 10 and 12.

## Discussion

### Relationship between the void ratio and shear displacement

The displacements significantly increased just prior to failure in most cases, as examined in the previous section, and deformation just prior to failure could not be distinguished well in the temporal variation in the displacements. Deformation behaviour just prior to failure can be scoped by adopting average SD instead of time. Thus, the relationship between the average SD and the void ratio, average VWC, is examined here to observe the behaviour well just prior to failure.

**Figure 11:**
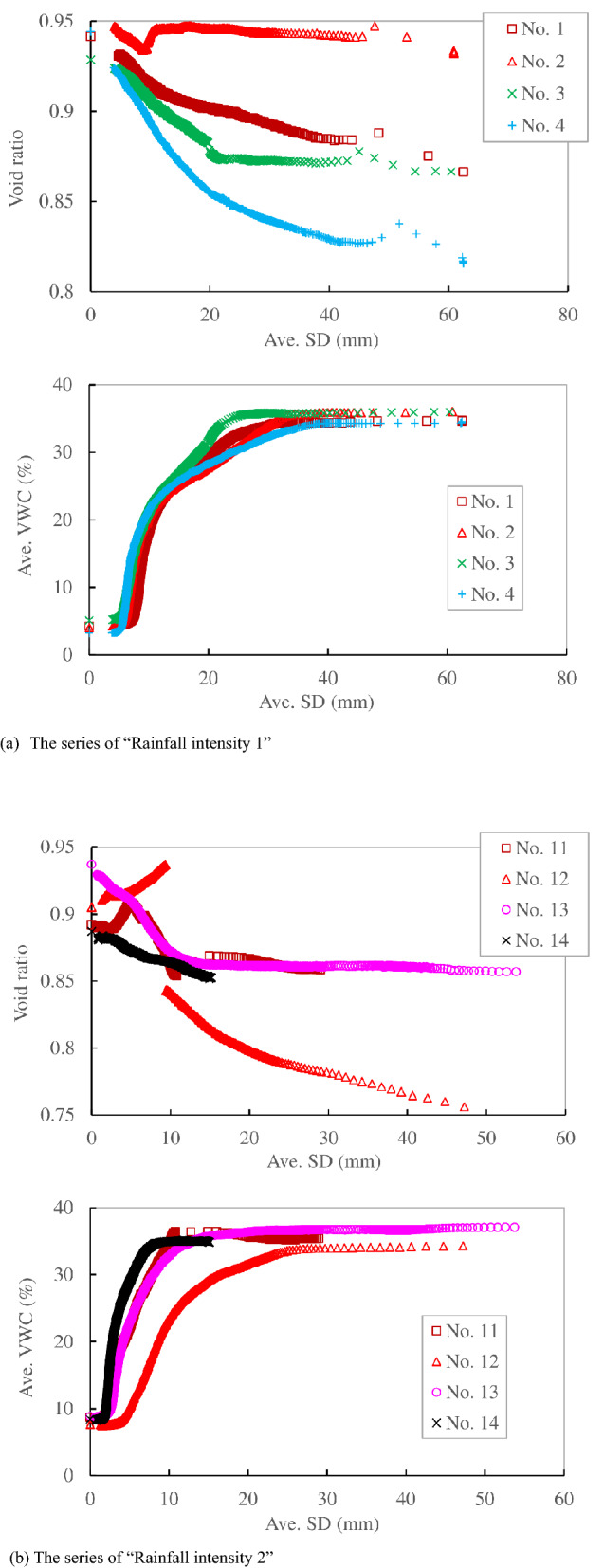

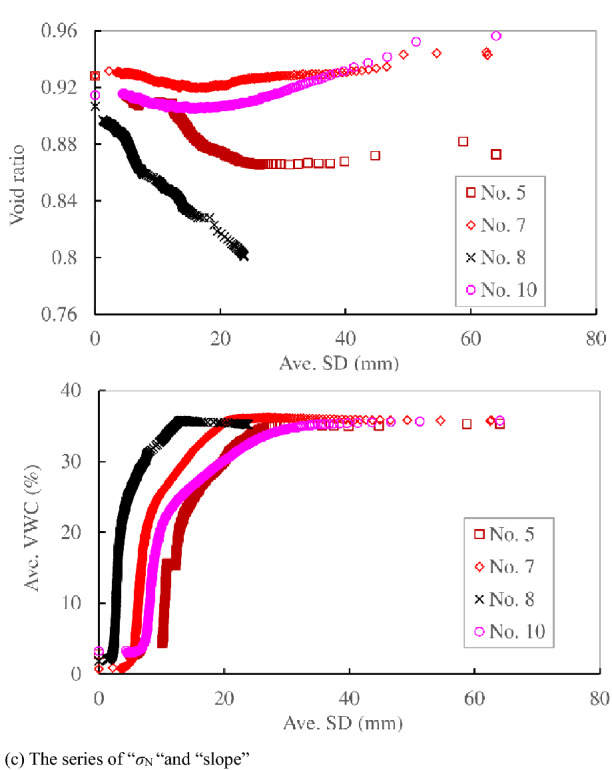
Comparison of the relationship between the average surface displacement (Ave. SD) and void ratio, average volumetric water content (Ave. VWC) in the series of “Rainfall intensity 1”, “Rainfall intensity 2”, “*σ*_N_ “and “slope”.

Figure 11a shows the relationship between the average SD and void ratio, average VWC in the series of “Rainfall intensity 1”. The shear displacement proceeded under constant VWC just prior to failure in Nos. 1 and 3, while it increased up to failure with a monotonic increase in VWC in Nos. 2 and 4. The initial VWC at each test was 4–5.1%, which is almost identical. The average VWC was almost identical until 16 mm of average SD, while it varied among the cases until 35 mm. The average VWC reached a constant VWC (35%) at 25 mm of average SD in No. 3, while it reached a constant VWC after 35 mm of average SD in the other cases. The average SD proceeded from 35 mm to failure under a constant average VWC in all cases, which could not be recognized in the relationship between time and average SD. It is hypothesized that the variation in the VWC could not follow the rapid increase in the displacement during failure in this region. Therefore, the displacement under constant VWC in this region could not be recognized as creep. The specimens compressed according to the development of the average SD, while the compression, which was defined as the decrease in void ratio, was larger at a lower initial void ratio on average. The void ratio decreased monotonically with increasing average SD until 39 mm and then remained almost constant until failure at 43 mm under constant VWC in specimen No. 1. It also decreased monotonically with the increase in average SD until 22 mm and then stayed almost constant until failure at 43 mm under constant VWC in specimen No. 3. The converged void ratio just prior to failure was 0.87–0.88. The void ratio decreased and increased until 10 mm of average SD at first, and then it showed a slight decrease until failure at 45 mm in No. 2. The specimen compressed with the increase in average SD until 40 mm, and then the void ratio converged to constant after that under constant VWC in No. 4. The ultimate void ratios in Nos. 2 and 4 were 0.94 and 0.83, respectively. The constant void ratio under a constant VWC in Nos. 2 and 4 was recognized as the rapid deformation of the specimen without a subsequent decrease in the VWC during failure, as explained in this section.

Figure 11b shows the relationship between the average SD and void ratio, average VWC in the series of “Rainfall intensity 2”. The shear displacement proceeded under constant VWC just prior to failure in Nos. 11 and 13, while it increased up to failure with a monotonic increase in VWC in No. 12. The shear displacement terminated under constant VWC without reaching failure in No. 14. The initial void ratio, model slope inclination and normal stress were almost the same as those of the series of “Rainfall intensity 1”, while the initial water content and rainfall intensity were higher than those of “Rainfall intensity 1” in these cases. The average VWC significantly increased until 10 mm of average SD and then decreased slightly and converged to constant (36%) at 20 mm of average SD in No. 11. The average VWC significantly increased at first, and then the rate of the increase in the VWC decreased to converge to a constant VWC (36%) at 20 mm of average SD in No. 13. The average VWC significantly increased at first and then converged to a constant value (35%) at 8 mm of average SD in No. 14. It significantly increased at first, and then the rate of increase gradually decreased, finally reaching a constant (34%) at 24 mm of average SD in No. 12, which was recognized as the start of failure because the increase in average SD was much larger than in the previous stage. The void ratio showed an increase and decrease until 10 mm of average SD and then slightly decreased to converge to a constant value (0.86) at 25 mm of an average SD under a constant VWC SD in No. 11. The void ratio monotonically decreased until 10 mm of average SD and remained constant after that in No. 13. The constant void ratio was almost the same (0.86–0.87) just prior to failure in these cases with creep deformation, and it was almost the same as the cases of surface displacement increase under constant VWC in Nos. 1 and 3 in the series of “Rainfall intensity 1”. The void ratio increased at first until an average SD of 9 mm and then significantly decreased until failure in No. 12. The void ratio monotonically decreased until the termination of the average SD in No. 14.

Figure 11c shows the relationship between the average SD and void ratio, average VWC in the series of “*σ*_N_ “and “slope”, which had different stress conditions to “Rainfall intensity 1” and “Rainfall intensity 2”. The void ratio decreased almost linearly with increasing average SD in No. 8, which did not reach failure under a constant VWC. It slightly decreased (compressed) at first and then increased (dilated) in No. 7 and No. 10 with higher normal stress and higher slope inclination. The specimen of No. 10 with higher slope inclination showed larger dilation just prior to failure while the specimen of No. 7 with higher normal stress showed very slight dilation just prior to failure. The specimens at higher stress levels showed dilation just prior to failure, such as in Nos. 7 and 10, while the specimens at lower stress conditions showed the trend of compression, such as in Nos. 5 and 8. The specimen was significantly compressed at first, and then the rate of compression gradually decreased in No. 5 under increasing VWC. This was the same behaviour as that of No. 4 and No. 12, which failed under increasing VWC. The slight decrease and increase in void ratio at around 0.93 just prior to failure in No. 5 and No. 7, respectively, can be recognized as the quasi-constant void ratio under constant VWC. The ultimate void ratio just prior to failure (0.93) in these cases with higher stress conditions was different from the value (0.86–0.87) in “Rainfall intensity 1” and “Rainfall intensity 2” with lower stress levels.

## Conclusion

Water was supplied to the specimen made of sandy soil in an inclined shear box, and shear and normal displacements were measured to examine the possibility of evaluating the condition of the onset of failure based on the multi-dimensional displacement. The results were derived as follows.

1. The shear displacement showed accelerative increase just prior to failure in most cases while it terminated without failure in some of the cases. Higher rainfall intensity produced faster failure time with the compression along the shear displacement. Normal stress did not influence on the time variation of the shear displacement while the model slope inclination gave large influence on the time variation of the shear displacement. The specimen tended to compress under lower normal stress and model slope inclination while it tended to dilate under high normal stress and model slope inclination.
2. The shear behaviour of the specimen with the increase in VWC was categorized into three cases. The first was that shear displacement increased up to failure with increasing VWC. The second was the increase in shear displacement up to failure with constant VWC just prior to failure. The third was that shear displacement increased at first and then terminated under constant VWC.
3. The constant VWC after the increase followed the constant suction after the decrease according to water supply to the specimen. Thus, a constant VWC after the increase suggested constant suction. The shear displacement increase under constant VWC was recognized as creep deformation under constant stress conditions.
4. Shear displacement increased up to failure with the monotonic decrease in void ratio (compression) in the case of approaching failure with increasing VWC under lower stress conditions. The specimen was slightly compressed at first and then dilated with increasing VWC under higher stress conditions.
5. Void ratio monotonically decreased under constant VWC in the case of shear displacement termination under constant VWC.
6. The void ratio significantly decreased at first and then converged to a constant just prior to failure in the case of shear displacement under constant VWC just prior to failure. The converged void ratio was 0.87–0.88 under the condition of slope inclination of 30 (deg), *σ*_N_ of 8.61 (kN/m^2^), while the ultimate void ratio under higher stress conditions was different from the value.
7. The void ratio or change in volume of the specimen can be recognized as an indicator of the failure of specimen under creep deformation under constant stress conditions according to absorption. When the void ratio tends to converge to a constant value, the shear displacement might increase up to failure. While the void ratio still varies under constant VWC, it might suggest that the shear displacement terminates before failure.

## Data Availability

The datasets used and/or analysed during the current study available from the corresponding author on reasonable request.
